# Spectral Imprints of Working Memory for Everyday Associations in the Frontoparietal Network

**DOI:** 10.3389/fnsys.2018.00065

**Published:** 2019-01-08

**Authors:** Elizabeth L. Johnson, David King-Stephens, Peter B. Weber, Kenneth D. Laxer, Jack J. Lin, Robert T. Knight

**Affiliations:** ^1^Helen Wills Neuroscience Institute, University of California, Berkeley, Berkeley, CA, United States; ^2^Institute of Gerontology, Wayne State University, Detroit, MI, United States; ^3^Department of Neurology and Neurosurgery, California Pacific Medical Center, San Francisco, CA, United States; ^4^Comprehensive Epilepsy Program, Department of Neurology, University of California, Irvine, Irvine, CA, United States; ^5^Department of Biomedical Engineering, University of California, Irvine, Irvine, CA, United States; ^6^Department of Psychology, University of California, Berkeley, Berkeley, CA, United States

**Keywords:** working memory, prefrontal cortex, parietal cortex, oscillations, directional connectivity, iEEG, ECoG

## Abstract

How does the human brain rapidly process incoming information in working memory? In growing divergence from a single-region focus on the prefrontal cortex (PFC), recent work argues for emphasis on how distributed neural networks are rapidly coordinated in support of this central neurocognitive function. Previously, we showed that working memory for everyday “what,” “where,” and “when” associations depends on multiplexed oscillatory systems, in which signals of different frequencies simultaneously link the PFC to parieto-occipital and medial temporal regions, pointing to a complex web of sub-second, bidirectional interactions. Here, we used direct brain recordings to delineate the frontoparietal oscillatory correlates of working memory with high spatiotemporal precision. Seven intracranial patients with electrodes simultaneously localized to prefrontal and parietal cortices performed a visuospatial working memory task that operationalizes the types of identity and spatiotemporal information we encounter every day. First, task-induced oscillations in the same delta-theta (2–7 Hz) and alpha-beta (9–24 Hz) frequency ranges previously identified using scalp electroencephalography (EEG) carried information about the contents of working memory. Second, maintenance was linked to directional connectivity from the parietal cortex to the PFC. However, presentation of the test prompt to cue identity, spatial, or temporal information changed delta-theta coordination from a unidirectional, parietal-led system to a bidirectional, frontoparietal system. Third, the processing of spatiotemporal information was more bidirectional in the delta-theta range than was the processing of identity information, where alpha-beta connectivity did not exhibit sensitivity to the contents of working memory. These findings implicate a bidirectional delta-theta mechanism for frontoparietal control over the contents of working memory.

## Introduction

The ability to maintain and manipulate information in working memory provides the neurobiological infrastructure for thinking and complex cognition. For 80 years, dominant views of working memory have emphasized the key role of the prefrontal cortex (PFC; Szczepanski and Knight, 2014). However, we previously demonstrated that working memory for everyday associations depends on frequency multiplexing between prefrontal and parieto-occipital regions (Johnson et al., 2017). Delta-theta (2–7 Hz) rhythms were observed in the PFC → parieto-occipital direction in response to a shift in task demands imposed by retrocuing identity, spatial, or temporal information from working memory stores. This oscillatory response was attenuated in individuals with focal lesions to the lateral PFC, resulting in a mean decrease of 8% in task accuracy. In contrast, parallel alpha-beta (9–24 Hz) rhythms were observed in the parieto-occipital → PFC direction, and were neither responsive to shifts in task demands nor affected by PFC lesions, revealing an oscillatory response that was independent of the PFC. These findings challenge dominant models of working memory which attribute function to PFC-dependent systems (Goldman-Rakic, 1995; Miller and Cohen, 2001; Curtis and D’Esposito, 2003; Müller and Knight, 2006; Lara and Wallis, 2014; Sreenivasan et al., 2014; Szczepanski and Knight, 2014; D’Esposito and Postle, 2015; Eriksson et al., 2015), and instead support a model of network-wide frontoparietal control (Wager and Smith, 2003; Duncan, 2010, 2013; Niendam et al., 2012; Cole et al., 2013; Ester et al., 2015, 2016; Sadaghiani and Kleinschmidt, 2016; Christophel et al., 2017).

The frontoparietal network is posited to govern the cascade of attentional processes that underlie complex cognitive functions and fluid intelligence (Duncan, 2010, 2013; Stoewer et al., 2010), including but not limited to working memory. This proposal draws evidence from studies showing flexible coding of task-specific events within prefrontal and parietal regions (Fusi et al., 2016; Stokes et al., 2017), including rapid, task-relevant changes in broadband gamma and higher-frequency power spectra in humans (Guillem et al., 1996; Miller et al., 2014) and single-unit activity in macaques (Balaguer-Ballester et al., 2011; Rigotti et al., 2013; Stokes et al., 2013). Moreover, evidence for these regions acting in concert comes from studies showing robust long-range anatomical tracts between frontal and parietal regions (Goldman-Rakic, 1988; Selemon and Goldman-Rakic, 1988; Cavada and Goldman-Rakic, 1989; Cabeza et al., 2008), as well as functional MRI studies showing that frontoparietal network connectivity is more sensitive to the demands imposed by a given cognitive task than that of other functional networks (Niendam et al., 2012; Cole et al., 2013). Finally, studies conducted in macaques indicate that the strength of frontoparietal network oscillatory synchrony carries information about items stored in working memory (Salazar et al., 2012; Dotson et al., 2014; Antzoulatos and Miller, 2016; Jacob et al., 2018). However, the oscillatory mechanisms of frontoparietal control over the contents of working memory are largely unexplored in humans.

**Figure 1:**
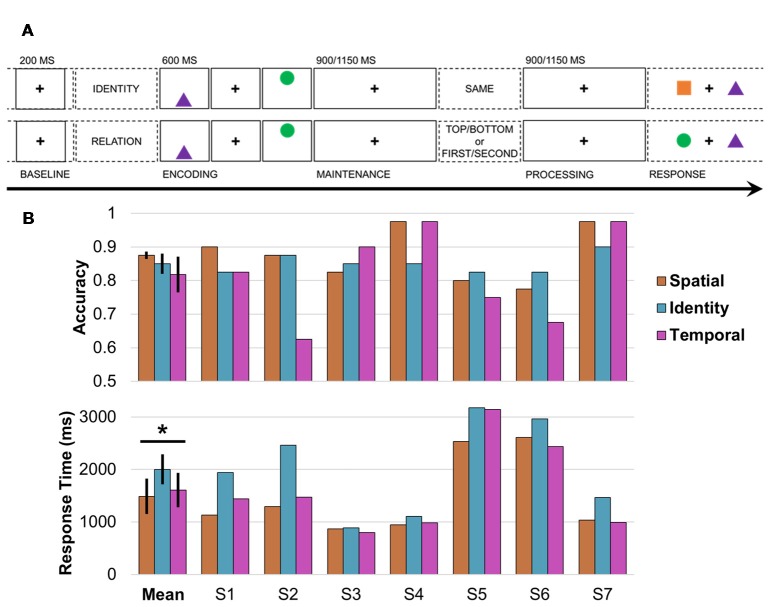
Working memory task design and behavior. **(A)** Single-trial working memory task design. Following a 1-s pretrial fixation interval (−250 to −50 ms pretrial epoch used as baseline), subjects were directed to focus on either IDENTITY or RELATION information. Then, two common shapes were presented for 200 ms each in a specific spatiotemporal configuration (i.e., top/bottom spatial and first/second temporal positions). After a 900- or 1,150-ms jittered pre-cue fixation delay (“maintenance”), the test cue appeared (i.e., one word presented on screen for 800 ms), followed by a post-cue fixation delay of the same length (“processing”). Working memory was tested in a two-alternative forced choice test (0.5 chance rate). In the identity test (top), subjects indicated whether the pair was the SAME pair they just studied (correct response in this example: no). In the spatiotemporal relation test (bottom), subjects indicated which shape fit the TOP/BOTTOM spatial or FIRST/SECOND temporal relation cue (correct response for cue TOP or SECOND: circle). **(B)** Mean and per-subject task accuracy (top) and response time (RTs; bottom). *Significant result; error bars, SEM; S, subject; orange, spatial trials; blue, identity trials; pink, temporal trials.

The purpose of this study was to investigate how the frontoparietal network supports the flexible coding of task-specific, ecologically valid events in humans. Simultaneous prefrontal and parietal recordings were obtained directly from the cortices of seven young adults while they performed a visuospatial working memory task that operationalizes the types of associations we encounter every day. Each trial was comprised of five phases: pretrial, encoding, pre-cue delay (“maintenance”), post-cue delay (“processing”), and response (Figure 1A; Johnson et al., 2017, 2018). Following the pretrial central fixation and start screen, two common shapes were presented sequentially in a top/bottom spatial orientation. A test cue was then presented mid-delay to retroactively cue specific information about the items being maintained in working memory: SAME (identities; Figure 1A, top), TOP/BOTTOM [spatial relations (bottom)], or FIRST/SECOND [temporal relations (bottom)]. This critical manipulation permitted us to examine how working memory unfolded over time as task demands shifted within a trial (i.e., maintenance vs. processing), and compare activity during the selection of identity vs. spatial or temporal information between trials. Because the maintenance of information about shape identity was common to all trials (Manohar et al., 2017), identity trials provided an exemplary control condition against which to contrast working memory for spatial and temporal information.

We hypothesized that delta-theta oscillations would: (1) be responsive to within-trial shifts in working memory demands, replicating previous work using this task (Johnson et al., 2017, 2018); and (2) differ between trials as a function of the specific information being selected in working memory. Further, if frontoparietal delta-theta oscillations support the flexible coding of task-specific events, then these rhythms will exhibit sensitivity to both within- and between-trial shifts in working memory task demands (Duncan, 2010, 2013). The use of intracranial electroencephalography (iEEG/ECoG) recordings provided a method to assess the per-trial spatial distribution of local and directional delta-theta effects (Johnson and Knight, 2015; Parvizi and Kastner, 2018), and—importantly—test alternative outcomes outside the delta-theta frequency range.

## Materials and Methods

### Subjects

We report data from seven adults, mean ± SD: 25.4 ± 3.3 years of age (four males), who were undergoing intracranial monitoring as part of seizure management. Electrodes were implanted solely on the clinical needs of each patient, and we selected datasets for inclusion *via* off-site review of individual neuroanatomical coverage. These datasets were collected at 4 sites: University of California (UC), Irvine, Hospital (three subjects with subdural and/or stereotactic implants); California Pacific Medical Center (CPMC; two subjects with subdural implants); UC San Francisco, Hospital (one subject with subdural implants); or Oslo University Hospital (one subject with stereotactic implants). This study was approved by and carried out in accordance with the recommendations of the Institutional Review Board of UC Berkeley, CPMC, UC San Francisco, or Regional Committee for Medical Ethics, Region South. All subjects gave written informed consent in accordance with the Declaration of Helsinki.

### Behavioral Task

Working memory was tested in a single-trial task paradigm that has been used previously to study neural networks in patient samples (Figure 1A; Johnson et al., 2017, 2018). After each 1-s pretrial fixation interval, a starting screen (800 ms) indicated whether the upcoming pair of stimuli would be tested for IDENTITY or spatiotemporal RELATION information. Then, following a 100-ms central fixation, two common-shape stimuli were presented for 200 ms each in a specific spatiotemporal configuration (i.e., top/bottom spatial and first/second temporal positions). The test cue was presented (800 ms) after a 900- or 1,150-ms delay interval to elicit information-specific selection mechanisms during a second delay interval of the same length. The length of the delays was randomly jittered to preclude anticipatory mechanisms. Then, two shapes were presented on the horizontal axis and subjects responded in a two-alternative forced choice test, resulting in a 0.5 chance rate. In the identity test, subjects indicated whether the pair was the SAME pair they just studied; half of the pairs show two old shapes (“yes”) and half the pairs show one old shape and one new shape (“no”), using the up and down arrow keys. In the spatial relation test, subjects indicated which shape had been on the TOP or BOTTOM, and in the temporal relation test, which shape had been presented FIRST or SECOND, using the left and right arrow keys.

The task was fully counterbalanced with 120 trials divided evenly between identity, spatial, and temporal conditions, chosen randomly from a pool of 150 trials with unique stimuli. An experimenter went through the experimental task instructions and a set of six practice trials with each subject, who was permitted to repeat the practice trials by request. All subjects completed the working memory task. The task was programmed in E-Prime Professional 2.0 (Psychology Software Tools, Pittsburgh, PA, USA).

Accuracy and correct-trial response time (RT) data were submitted to logit and linear mixed-effects models, respectively, with three condition fixed effects and seven subject random effects (Cramer et al., 2015). In the case of a significant outcome, *post hoc* testing was repeated between each pair of conditions.

### Electrode Localization

**Figure 2:**
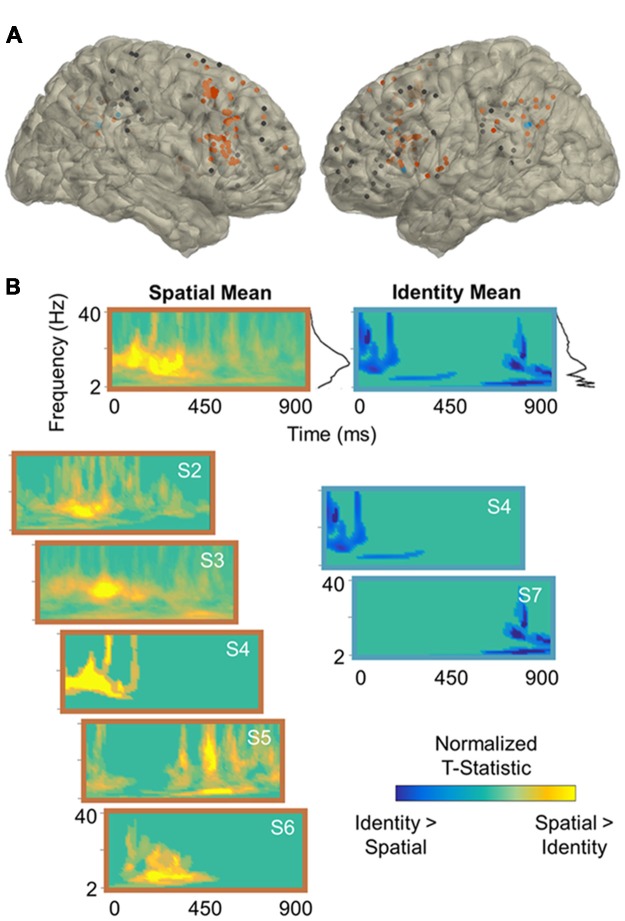
Spatial processing power effects. **(A)** Reconstruction of frontoparietal electrode coverage for all subjects. Electrodes are color-coded according to the results of the per-subject contrast between identity and spatial trials: orange, spatial > identity; blue, identity > spatial; black, no effect. All effects are significant at the cluster-corrected threshold of 0.05. **(B)** Mean and per-subject time-frequency representations of significant *t*-statistics (cluster-corrected mask applied) for the electrodes indicated in **(A)**. The black lines to the right of the means indicate the spectral density of the corresponding time-frequency representations (i.e., mean significant *t*-values per frequency). Frequencies range from 2 Hz to 40 Hz in linearly-spaced steps of 1 Hz. *T-statistics* are normalized according to the maximum value of each plot (scale: −1 to +1) and color-coded by the direction of effects: cooler, identity > spatial; warmer, spatial > identity. S, subject.

**Figure 3:**
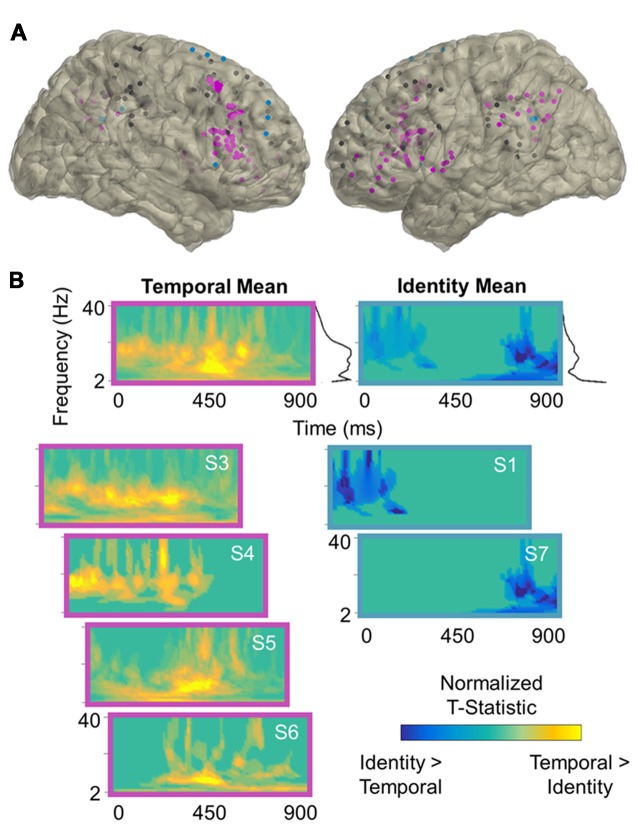
Temporal processing power effects. **(A)** Reconstruction of frontoparietal electrode coverage for all subjects. Electrodes are color-coded according to the results of the per-subject contrast between identity and temporal trials: pink, temporal > identity; blue, identity > temporal; black, no effect. All effects are significant at the cluster-corrected threshold of 0.05. **(B)** Mean and per-subject time-frequency representations of significant *t*-statistics (cluster-corrected mask applied) for the electrodes indicated in **(A)**. The black lines to the right of the means indicate the spectral density of the corresponding time-frequency representations (i.e., mean significant *t*-values per frequency). Frequencies range from 2 Hz to 40 Hz in linearly-spaced steps of 1 Hz. *T-statistics* are normalized according to the maximum value of each plot (scale: −1 to +1) and color-coded by the direction of effects: cooler, identity > temporal; warmer, temporal > identity. S, subject.

Electrodes were localized for each subject based on individual anatomy (Table 1), and then transferred into standard MNI space for presentation across subjects (Figures 2A, 3A). Affine point-based registration was used to co-register post-implantation computed tomography (CT) coordinates to the pre-operative magnetic resonance (MR) images using the FieldTrip toolbox (Oostenveld et al., 2011; Stolk et al., 2018) for MATLAB (MathWorks, Inc., Natick, MA, USA). Subjects were selected based on electrode placement covering both lateral prefrontal and parietal cortices.

**Table 1:** Individual electrode coverage.

	Total electrodes	Electrodes analyzed	Hemisphere
S1	72	PFC: 21	L + R
		Parietal: 2	L + R
S2	96	PFC: 24	L + R
		Parietal: 8	L + R
S3	256	PFC: 54	R
		Parietal: 14	R
S4	116	PFC: 12	L + R
		Parietal: 10	R
S5	172	PFC: 10	L
		Parietal: 16	L
S6	108	PFC: 8	L
		Parietal: 4	L
S7	101	PFC: 4	L
		Parietal: 5	L

*S, subject; L, left; R, right*.

### Data Acquisition and Preprocessing

UC Irvine data were acquired using a Nihon Kohden recording system, sampled at 5 or 10 kHz and resampled offline to 1 kHz. CPMC data were acquired using a Nihon Kohden recording system, sampled at 1 kHz. UC San Francisco data were acquired using a Tucker Davis Technologies recording system, sampled at 1.526 kHz and resampled offline to 1 kHz. Oslo data were acquired using a Nicolet (NicOne) recording system, sampled at 512 Hz. As described below, spectral decomposition was performed up to 40 Hz, and so 512 Hz is well over the minimum Nyquist frequency (2 cycles/frequency = 80 Hz) required for analysis. Preprocessing routines were performed using the FieldTrip (Oostenveld et al., 2011) and EEGLAB (Delorme and Makeig, 2004) toolboxes for MATLAB.

Raw electrophysiology data traces were manually inspected under the supervision of a neurologist (RTK), who was blinded to electrode locations and experimental task parameters. Channels and epochs displaying epileptiform activity or artifactual signal (from poor contact, machine noise, etc.), and those placed on tissue that was later resected were excluded from analysis. Remaining channels were filtered with 1-Hz high-pass and 200-Hz low-pass (165-Hz for Oslo data) finite impulse response filters and demeaned, and 60-Hz line noise harmonics (50-Hz for Oslo data) were removed using discrete Fourier transform. We re-inspected the filtered data to mark any channels or epochs containing residual artifacts for exclusion. Then, every artifact-free electrode within the prefrontal or parietal cortex was re-referenced to the next adjacent electrode, spaced at 4, 5, or 10 mm within that region, using bipolar montages to create virtual electrodes with minimized contamination from volume conduction (Shirhatti et al., 2016; Trongnetrpunya et al., 2016). The final dataset contained 192 virtual electrodes, range (mean) per subject: 4–54 (19) PFC, 2–16 (8) parietal cortex; see Table 1 for per-subject electrode localization information.

We then epoched the continuous data into trials with 1-s buffers (i.e., first stimulus onset −1 s to response screen onset +1 s) and excluded trials overlapping with epochs that had been marked as noisy during inspection, and again manually re-inspected the data to reject any trials with residual noise. The final dataset included an average of 96 correct trials per subject, mean ± SD (range) trials: 32 ± 3 (26–35) identity, 33 ± 4 (27–39) spatial relation, 31 ± 6 (20–39) temporal relation. There were too few incorrect trials for meaningful electrophysiological data analysis, mean ± SD (range) trials: 17 ± 8 (4–25) per subject. Finally, the data were epoched into three segments per trial for analysis (see Figure 1A): (1) 200-ms pretrial baseline interval extending from 250 ms to 50 ms before the start screen; (2) 900-ms pre-cue delay interval extending from the offset of the second stimulus (“maintenance”); and (3) 900-ms post-cue delay interval extending from the offset of the test cue (“processing”). The post-cue processing interval is the critical analysis interval because the subject was actively processing either identity, spatial, or temporal information in working memory (Figure 1A). As described below, the processing interval was analyzed separately for event-related potentials (ERPs), task-induced power, and directional connectivity.

### Event-Related Potentials

The correct-trial 200-ms pretrial baseline and 900-ms post-cue processing data segments were zero-padded to 7,500 ms to minimize filtering-induced edge artifacts and passed through a 30-Hz low-pass finite impulse response filter (Johnson et al., 2017, 2018). Task-induced ERPs were computed over the processing interval by absolute baseline-correcting the outputs on the temporal mean of the pretrial baseline (i.e., post-cue processing—pretrial mean).

The outputs were tested per-subject for condition differences between identity and spatial/temporal trials over the processing interval. Within-subjects statistical testing employed a Monte Carlo method (1,000 iterations) with cluster-based maximum correction for multiple comparisons (Maris and Oostenveld, 2007). An independent-samples *t*-test was used to identify clusters of contiguous data points showing a difference between conditions, thresholded at 0.05, two-tailed, and then the *t-statistics* were summed over all data points per cluster to calculate cluster size. Effects were clustered on the basis of spatial (i.e., neighboring electrodes) and temporal adjacency. Then, condition labels were randomly shuffled and the same clustering procedure was applied 1,000 times to create a normal distribution of null effects. Observed clusters were considered significant if fewer than 5% of randomizations yielded a larger effect (i.e., cluster-corrected *α* = 0.05). Statistical testing was performed using FieldTrip functions in MATLAB (Oostenveld et al., 2011).

### Spectral Decomposition

Time-frequency representations of power were computed on the correct-trial 200-ms pretrial baseline and 900-ms post-cue processing data segments. Data segments were zero-padded to 7,500 ms and time-frequency representations were computed using an adaptive, frequency-dependent sliding window of 3 cycles’ length (Δt = 3/*f*) for frequencies from 2 Hz to 40 Hz (1-Hz steps, 2-Hz bandwidth). The time windows were advanced in steps of 10 ms and the data in each window were multiplied with a Hanning taper before calculating power using fast Fourier transforms. For a similar approach, see Johnson et al. (2017).

Task-induced power was analyzed per subject and trial using a statistical bootstrapping procedure. Baseline power values were pooled into a single time-series for each channel and frequency, from which we randomly selected and averaged *r* data points (*r* = number of trials in that subject’s dataset). This step was repeated 1,000 times to create normal distributions of electrode and frequency-resolved pretrial baseline data. Delay raw power data were z-scored on the pretrial baseline distributions to assess the significance of task-induced effects. For a similar approach, see Flinker et al. (2015); Johnson et al. (2017) and Johnson et al. (2018).

Within-subjects statistical testing of condition differences between identity and spatial/temporal trials was equivalent to that for ERPs, with clustering on the electrode, time, and frequency dimensions.

### Directional Connectivity

Directional connectivity was computed on the correct-trial 900-ms pre-cue maintenance and post-cue processing data segments between signals across each PFC-parietal cortex electrode pair within the same hemisphere using the Phase Slope Index (PSI; Nolte et al., 2008). The PSI metric tracks whether the slope of the phase lag between A and B electrode pairs is consistent across several adjacent frequency bins. Positive PSI indicates that electrode A leads electrode B, negative PSI indicates the reverse, and zero PSI indicates either zero or an evenly balanced lead/lag relationship between electrodes.

#### Task-Induced PSI

The raw trial-wise mean for correct-trial data segments was first subtracted from each raw correct-trial data segment. Spectral representations were then computed from the outputs using the same parameters described above, but over the full delay interval (i.e., not time-resolved). Importantly, the Hanning taper reduces spectral leakage and allows us to keep the bandwidth constant for computation of PSI. Cross-spectral density was calculated between the complex Fourier outputs, from which PSI was computed separately for the delta-theta (2–7 Hz) and alpha-beta (9–24 Hz) bands (Johnson et al., 2017).

Per-subject statistical analysis of PSI was performed for the delay intervals by standardizing the outputs against frequency-shuffled surrogate distributions *via* bootstrapping. At each electrode pair and frequency point, we randomly shuffled the frequencies in one signal and re-computed PSI 1,000 times to create normal distributions of electrode-pair and frequency-resolved null PSI data. Raw PSI outputs were z-scored on the null distributions to correct for any spurious results and assess the significance of directional effects. PFC leads were defined as PSI *z* > 1.96 and parietal leads as *z* < −1.96 (i.e., *α* = 0.05); for a similar approach, see Johnson et al., 2017, 2018. PSI data were visualized topographically using the BrainNet Viewer for MATLAB (Xia et al., 2013).

The outputs were submitted to group statistical testing using linear mixed-effects models with 2 task-interval fixed effects (i.e., maintenance vs. processing), and seven subjects and 1,129 electrode pairs as random effects (Johnson et al., 2018).

#### Time-Resolved PSI

We recomputed time-resolved PSI (10-ms resolution) on the post-cue processing data segments, per condition, for all pairs of electrodes that exhibited significant directionality (PSI |*z|* > 1.96) over the whole processing interval (Johnson et al., 2018). Spectral decomposition, PSI computation, and per-subject statistical bootstrapping analysis were otherwise identical. By including only directional electrode pairs, this step ensures that the interpretation of output PSI values close to zero (i.e., |*z*| < 1.96, *p* > 0.05) would be unambiguous—that is, evidence of evenly balanced lead/lag relationships rather than zero relationship.

The outputs were submitted to group-level statistical testing by sliding the linear mixed-effects model across timepoints. At each 10-ms timepoint, there were two condition fixed effects (identity vs. spatial/temporal), and seven subject and 1,008 (in delta-theta models) or 984 (alpha-beta models) electrode-pair random effects. The outputs were corrected for multiple comparisons on the time dimension using the false discovery rate (FDR) threshold of 0.05. Significant differences were considered sustained if they persisted for at least 100 ms at the FDR-corrected threshold (e.g., Flinker et al., 2015; Foo et al., 2016).

### Data Availability

The data and custom-built MATLAB codes that support the current findings are deposited to the University of California, Berkeley, Collaborative Research in Computational Neuroscience database^1^.

## Results

### Behavior

We confirmed that all subjects were proficient at the task (accuracy range 0.63–0.98, chance 0.5; Figure 1B, top), and then submitted the accuracy and RT data to mixed-effects models with three condition fixed effects and seven subject random effects. There was a significant effect of condition on RT (*F*_(1,19)_ > 6.50, *p* < 0.02, *d* > 1.47; Figure 1B, bottom), but no effect on accuracy (*p* > 0.50). *Post hoc* testing revealed that RTs were longer on identity than spatial (*F*_(1,12)_ > 13.78, *p* < 3 × 10^−3^, *d* = 2.14) and temporal (*F*_(1,12)_ > 10.95, *p* < 7 × 10^−3^, *d* = 1.91) trials, with no difference between spatial and temporal trials (*p* > 0.22).

### Cortical Representations

All frontoparietal electrodes (*n* = 192) were submitted to analyses of ERPs and task-induced power, and the outputs were tested per subject for condition differences between identity and spatial/temporal trials (see Figure 1A) using non-parametric statistics with cluster-based correction for multiple comparisons (Maris and Oostenveld, 2007). We investigated working memory for space and time by comparing spectral activity during the selection of an item in space or time to the ongoing maintenance of item identity (i.e., spatial/temporal > identity effects; Johnson et al., 2018). We further considered sensitivity to task difficulty in cases where those subjects who exhibited both increased RTs and decreased accuracy on identity trials also exhibited identity > spatial/temporal spectral effects.

#### ERPs

ERPs were quantified (1–30 Hz bandpass) over the 200-ms pretrial baseline and 900-ms post-cue processing intervals for correct trials (see Figure 1A), and then processing outputs were absolute baseline-corrected on the temporal mean of the pretrial baseline. Cluster-based permutation testing of identity vs. spatial/temporal trials indicated that ERPs did not differ between conditions in any subject (all *p* > 0.10), ensuring that spectral condition effects were not due to exogenous activity from ERPs (Johnson et al., 2018).

#### Spectral Effects

We then examined the spatio-spectral distributions of condition effects. Time-frequency representations of power were quantified from 2 Hz to 40 Hz (1-Hz steps, 2-Hz bandwidth) for the pretrial baseline and post-cue processing intervals for correct trials, and then processing outputs were standardized on the pretrial baseline *via* statistical bootstrapping (Flinker et al., 2015; Johnson et al., 2017, 2018).

Per-subject cluster-based permutation testing revealed that information about the contents of working memory was distributed throughout the frontoparietal network (all *p* < 0.04, cluster-corrected), with the majority of frontoparietal sites exhibiting sensitivity to working memory for spatiotemporal relationships between 2 Hz and 40 Hz. In the contrast between identity and spatial trials (Figure 2), 5/7 (71%) subjects showed significant spatial > identity effects (mean ± SD: 54 ± 22% of electrodes/subject) and 2/7 (29%) showed identity > spatial effects (36 ± 12% of electrodes/subject). In the contrast between identity and temporal trials (Figure 3), 4/7 (57%) subjects showed significant temporal > identity effects (68 ± 30% of electrodes/subject) and 2/7 (29%) showed identity > temporal effects (37 ± 10% of electrodes/subject). Importantly, the subjects who exhibited significant identity > spatial/temporal effects were the same subjects who exhibited both increased RTs and decreased accuracy on identity trials, suggesting that sparsely distributed frontoparietal sites are sensitive to task difficulty.

To examine the spectral components of working memory for space, we utilized the per-subject cluster-thresholded mask to index the power data points that showed significant spatial > identity processing and then averaged them across all electrodes per subject (Figure 2B, left). We repeated this procedure to examine the spectral components for time (temporal > identity processing; Figure 3B, left), as well as difficulty (identity > spatial/temporal processing; Figures 2B, 3B, right). Statistically significant effects were most prominent at lower frequencies, regardless of the exact contrast. Specifically, information about the contents of working memory appeared to be carried at distinct delta-theta (2–7 Hz) and alpha-beta (9–24 Hz) frequency ranges. These are the same frequency ranges previously identified as prefrontal- vs. parieto-occipital-led using scalp EEG on the same working memory task (Johnson et al., 2017).

### Directional Connectivity

PSI (Nolte et al., 2008) was computed between signals across each PFC-parietal cortex pair within the same hemisphere (*n* = 1,129) separately for the delta-theta (2–7 Hz) and alpha-beta (9–24 Hz) frequency ranges. PSI was first computed over the full 900-ms pre-cue maintenance and post-cue processing intervals (see Figure 1A) and not time-resolved. Each subject’s outputs were z-scored against frequency-shuffled reference distributions to assess directionality and then submitted to group-level testing as a function of task interval (Johnson et al., 2017, 2018).

Time-resolved PSI was re-computed per condition over the post-cue processing interval at all electrode pairs that were found to be significantly directional overall during the processing interval (*n* = 1,008 delta-theta, 984 alpha-beta). By including only directional electrode pairs, this step ensures that net output PSI values close to zero (i.e., |*z*| < 1.96, *p* > 0.05) would be considered evidence of bidirectionality. Each subject’s PSI outputs were z-scored per time point and then tested on the group level for differences between identity and spatial/temporal conditions.

#### Task-Induced Effects

**Figure 4:**
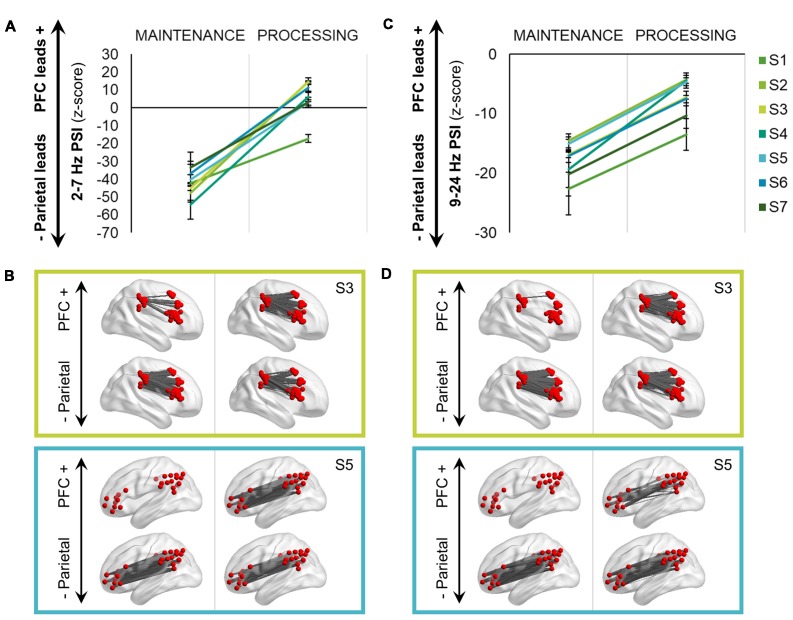
Bidirectional frontoparietal oscillations for information processing. **(A)** Delta-theta (2–7 Hz) phase slope index (PSI) shifted from a unidirectional, parietal-led network during maintenance to a bidirectional, frontoparietal network during the processing interval (*p* < 8 × 10^−306^). Data are represented as mean ± SEM per subject across all trials; positive values indicate that the prefrontal cortex (PFC) leads the parietal cortex and negative values indicate that the parietal cortex leads the PFC. S, subject. **(B)** Topographical representations of the PSI data depicted in **(A)** in two subjects. PSI is masked per electrode pair, with significant PFC leads in the top row (*z* > 1.96, *p* < 0.05) and parietal leads (*z* < −1.96, *p* < 0.05) in the bottom row. S3 was implanted subdurally in the right hemisphere and S5 in the left hemisphere. **(C)** Equivalent to **(A)**: alpha-beta (9–24 Hz) PSI shifted from a unidirectional, parietal-led network during maintenance to a bidirectional, but still net parietal-led network during the processing interval (*p* = 0). **(D)** Equivalent to **(B)**: topographical representations of the PSI data depicted in **(C)** in two subjects.

The maintenance interval was characterized by unidirectional parietal → PFC connectivity in both frequency ranges (threshold *z* < −1.96, *p* < 0.05), mean ± SD, delta-theta: *z* < −40.03 ± 8.73; alpha-beta: *z* < −30.37 ± 2.60. However, following presentation of the test cue to select identity, spatial, or temporal information from working memory stores, delta-theta directionality shifted from a unidirectional to bidirectional frontoparietal network (processing > maintenance, *F*_(1,2256)_ = 1936.10, *p* < 8 × 10^−306^; Figures 4A,B). Within individual subjects, some electrodes shifted in direction from parietal to PFC leads, while others decreased in the strength of parietal lead or did not change, reflecting spatially diverse patterns of bidirectional delta-theta connectivity during information processing (see Johnson et al., 2018). Importantly, the processing interval was characterized by net connectivity greater than zero in 6/7 subjects (86%), mean ± SD: *z* > 6.01 ± 6.42, revealing a network-wide shift from parietal-led to frontoparietal in the delta-theta range with working memory task demands (see Johnson et al., 2017).

All analyses were repeated for PSI in the alpha-beta range. In the alpha-beta range, there was again a shift in directionality with presentation of the test cue (processing > maintenance, *F*_(1,2256)_ = 2302.30, *p* = 0; Figures 4C,D). Despite the shift, however, all subjects continued to exhibit net parietal → PFC connectivity at processing, mean ± SD: *z* < −12.60 ± 7.51 (see Johnson et al., 2017).

#### Spatiotemporal Processing Effects

**Figure 5:**
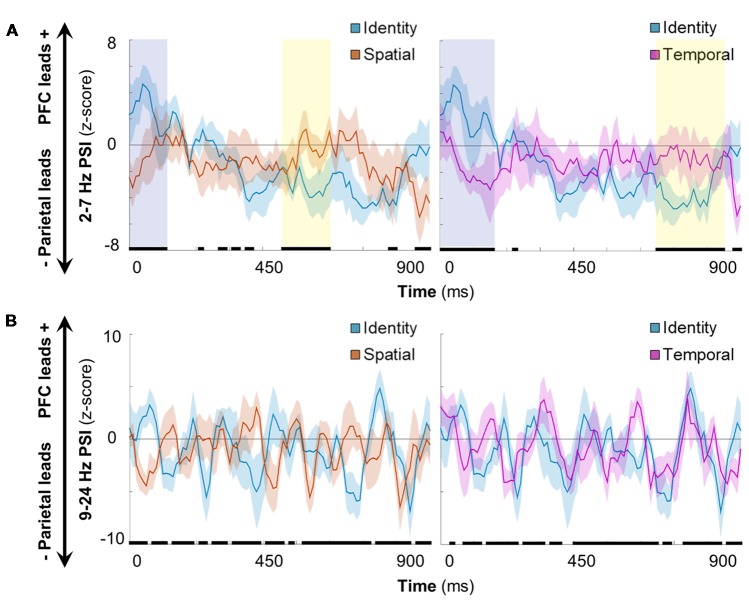
Spatiotemporal processing connectivity effects. **(A)** Grand mean time-resolved delta-theta PSI over the processing interval for the contrast between identity and spatial (left)/temporal (right) conditions. Data are represented as mean ± SEM across subjects; positive values indicate that the PFC leads the parietal cortex and negative values indicate that the parietal cortex leads the PFC. Black marks indicate the timepoints of significant condition effects at the false discovery rate (FDR)-corrected threshold of 0.05. Shaded epochs represent effects that were sustained for >100 ms, color-coded by the direction of effects: cooler, identity > spatial/temporal; warmer, spatial/temporal > identity. Orange, spatial trials; blue, identity trials; pink, temporal trials. **(B)** Equivalent to **(A)**: alpha-beta PSI did not show sustained effects for one condition or the other in either contrast.

Having established that distributed frontoparietal sites coded the contents of working memory (see Figures 2, 3) and that information processing elicited bidirectional frontotemporal interactions (Figure 4), we proceeded to examine rapid, time-resolved network interactions as a function of condition. We observed that delta-theta frontoparietal network connectivity was sensitive to the contents of working memory (Figure 5A). In the contrast between identity and spatial conditions (left), significant differences were sustained at the beginning (0–120 ms) and early in the second half (470–610 ms) of the post-cue processing interval (FDR-corrected *p* < 0.05, marked in black). In the contrast between identity and temporal conditions (right), significant differences were again sustained at the beginning (0–170 ms) and in the second half (660–860 ms) of the processing interval (FDR-corrected *p* < 0.05).

Further examination of the early post-cue processing data (0–120/170 ms) indicated that the PFC led parietal sites during the ongoing maintenance of identity information (mean, identity > spatial: *z* > 2.62, *p* < 9 × 10^−3^; identity > temporal: *z* > 2.35, *p* < 0.02). Later in processing (470–610/660–860 ms), however, the parietal cortex led prefrontal sites (mean, identity < spatial: *z* < −3.04, *p* < 3 × 10^−3^; identity < temporal: *z* < −3.57, *p* < 4 × 10^−4^), revealing sub-second shifts in directionality during working memory for information about item identity.

In contrast, the selection of spatial (Figure 5A, left) and temporal (right) information exhibited bidirectionality throughout the processing interval (all |*z*| < 1.60, *p* > 0.11). Early in processing (shaded in yellow), this dichotomy demonstrates that the selection of spatiotemporal information was more parietal-led than that for the ongoing maintenance of information about item identity. Later in processing, we observed that the epoch corresponding to the selection of spatial information ended ~50 ms before the start of the epoch corresponding to that of temporal information [shaded in blue (left vs. right)]. These results suggest that working memory for spatial and temporal information is supported by relatively more PFC-led network interactions than for identity information, and that spatial and temporal information selection occurs serially in the delta-theta band. Taken together, these results indicate that delta-theta rhythms exerted bidirectional control between prefrontal and parietal cortices during working memory, coding the contents of working memory with sub-second precision.

All analyses were repeated for PSI in the alpha-beta range. Unlike delta-theta directional connectivity, alpha-beta frontoparietal directionality switched rapidly between parietal-led and bidirectional over the course of the 900-ms post-cue processing interval (Figure 5B). Although there were significant differences between conditions (FDR-corrected *p* < 0.05, marked in black), the differences were not sustained for one condition or the other during either condition-pair contrast [i.e., identity vs. spatial conditions (left), identity vs. temporal conditions (right)]. Rather, it appears that these effects resulted from per-condition means that differed slightly in the timing of inter-regional connectivity. These results suggest that alpha-beta rhythms did not carry information about the contents of working memory on the network-level between prefrontal and parietal cortices.

## Discussion

Our findings demonstrate that low-frequency frontoparietal rhythms support the flexible coding of task-specific events in humans. Using an ecologically valid task of working memory for everyday “what,” “where,” and “when” associations (Johnson et al., 2017, 2018), we show that oscillations between 2 Hz and 24 Hz displayed sensitivity to both within- and between-trial shifts in task demands. First, per-subject analyses of task-induced power in the post-cue processing interval revealed that the majority of frontoparietal sites coded space and time, while a sparse minority also coded task difficulty (Figures 2, 3). This result indicates that spectral imprints of everyday associations in working memory are distributed throughout the frontoparietal network, supporting a network-level model of control (Duncan, 2010, 2013; Stoewer et al., 2010). Furthermore, we observed that this information was carried at distinct delta-theta (2–7 Hz) and alpha-beta (9–24 Hz) frequency ranges—the same frequency ranges previously identified as prefrontal- vs. parieto-occipital-led using scalp EEG on the same task (Johnson et al., 2017). We then proceeded to examine directional connectivity between prefrontal and parietal sites at each frequency range with the high spatial precision afforded by iEEG/ECoG recordings.

Second, analyses of per-subject directional connectivity revealed that maintenance of information in working memory was led by the parietal cortex. This result is consistent with proposals of mnemonic information storage in parietal regions (Bettencourt and Xu, 2015; Ku et al., 2015; Galeano Weber et al., 2017), upon which the PFC interacts in response to task demands (Miller and Cohen, 2001; Lara and Wallis, 2014; Sreenivasan et al., 2014; D’Esposito and Postle, 2015; Eriksson et al., 2015). However, we did not observe a reversal in directionality from parietal- to prefrontal-led with a shift in task demands, as would be expected under PFC-dependent control. Instead, we observed that presentation of the test prompt to retroactively cue and select specific information from working memory stores attenuated the parietal lead over the PFC (Figure 4). In the alpha-beta range, network connectivity remained parietal-led. In the delta-theta range, network connectivity exhibited a within-trial change from parietal-led to bidirectional frontoparietal coordination. These critical results support a network-wide model of control (Duncan, 2010, 2013; Stoewer et al., 2010), and reveal that bidirectional frontoparietal network connectivity is specific to the delta-theta range.

Third, analyses of time-resolved network connectivity revealed that spatiotemporal processing was linked to bidirectional delta-theta interactions (see Johnson et al., 2018), while the processing of identity information shifted from prefrontal- to parietal-led over the course of a 900-ms delay interval (Figure 5). This means that early in the post-cue processing interval, the coding of space and time was more parietal-led and later, that it was more prefrontal-led than that of the ongoing maintenance of identity information. Furthermore, we observed that space and time were processed serially in the PFC → parietal direction, suggesting that delta-theta rhythms flexibly code everyday spatiotemporal associations in quick succession. In contrast, we observed that alpha-beta connectivity was largely parietal-led and did not exhibit network-level, condition-specific coding of the contents of working memory.

These results are consistent with the hypothesis that frontoparietal delta-theta rhythms support the flexible coding of task-events, both locally and on the network level. However, we also observed that alpha-beta rhythms carried information about the contents of working memory at sites distributed across the frontoparietal network (see Salazar et al., 2012; Dotson et al., 2014; Antzoulatos and Miller, 2016; Jacob et al., 2018). These findings suggest that a range of low-frequency spectra carry transient imprints of everyday working memory associations at frontoparietal sites, while delta-theta oscillations selectively guide network communication according to task demands. The generalizability of the current findings is limited by the low number of incorrect trials, preventing direct comparison of correct and incorrect trials, and low number of subjects with simultaneous frontal and parietal cortical implants. However, as all statistical analyses were performed per subject prior to any group modeling, the consistency in results across subjects (Figures 2–4) indicates that the results are reliable.

Finally, we note that another group of iEEG patients displayed a similarly complex pattern of bidirectional interactions between the PFC and medial temporal lobe on the same working memory task, with exceptional patterns of bidirectional communication in the theta band (Johnson et al., 2018). Taken together, the results from these studies indicate that maintenance of information in working memory is linked to unidirectional delta-theta connectivity on a larger scale from parietal → prefrontal → medial temporal regions. Critically, flexible coding demands imposed by retrocuing specific “what,” “where,” or “when” information from working memory stores are sufficient to change the large-scale system from unidirectional to bidirectional, with delta-theta rhythmicity observed in both directions between the PFC and parietal and medial temporal regions during information selection. These findings build on recent work showing that frontoparietal networks are fundamentally rhythmic in nature, with delta-theta rhythms supporting attention-related activity in humans (Helfrich et al., 2018) and macaques (Fiebelkorn et al., 2018). They also apply to memory more generally, building on a recent finding that cued recall likewise fluctuates at theta frequencies in the human EEG (Kerrén et al., 2018).

We argue that, not only do delta and theta oscillations flexibly guide network control across frontoparietal regions, but also globally across multiple neural networks (see Cole et al., 2010) during working memory. These findings add to a growing body of literature proposing that low-frequency rhythms provide the infrastructure for neural communication supporting flexible cognitive processing.

## Author Contributions

EJ designed the study, analyzed the data, and wrote the manuscript. JL, DK-S, PW, and KL examined the patients. RK supervised the project and edited the manuscript.

## Conflict of Interest Statement

The authors declare that the research was conducted in the absence of any commercial or financial relationships that could be construed as a potential conflict of interest.

## Footnotes

^1^http://crcns.org
